# Graded or threshold response of the tet-controlled gene expression: all depends on the concentration of the transactivator

**DOI:** 10.1186/1472-6750-13-5

**Published:** 2013-01-22

**Authors:** Niels Heinz, Katharina Hennig, Rainer Loew

**Affiliations:** 1 EUFETS GmbH, Idar-Oberstein, 55743, Germany; 2 Experimental Haematology, Hannover Medical School, Hannover, 30625, Germany

**Keywords:** Tet-controlled gene expression, Transactivator concentration, Threshold response, Self-contained, Autoregulated

## Abstract

**Background:**

Currently, the step-wise integration of tet-dependent transactivator and tet-responsive expression unit is considered to be the most promising tool to achieve stable tet-controlled gene expression in cell populations. However, disadvantages of this strategy for integration into primary cells led us to develop an “All-In-One” vector system, enabling simultaneous integration of both components. The effect on tet-controlled gene expression was analyzed for retroviral “All-In-One” vectors expressing the M2-transactivator either under control of a constitutive or a new type of autoregulated promoter.

**Results:**

Determination of luciferase activity in transduced cell populations indicated improvement of the dynamic range of gene expression for the autoregulated system. Further differences were observed regarding induction kinetics and dose–response. Most notably, introduction of the autoregulated system resulted in a threshold mode of induction, whereas the constitutive system exhibited pronounced effector-dose dependence.

**Conclusion:**

Tet-regulated gene expression in the applied autoregulated system resembles a threshold mode, whereby full induction of the tet-unit can be achieved at otherwise limiting doxycycline concentrations.

## Background

**Figure 1:**
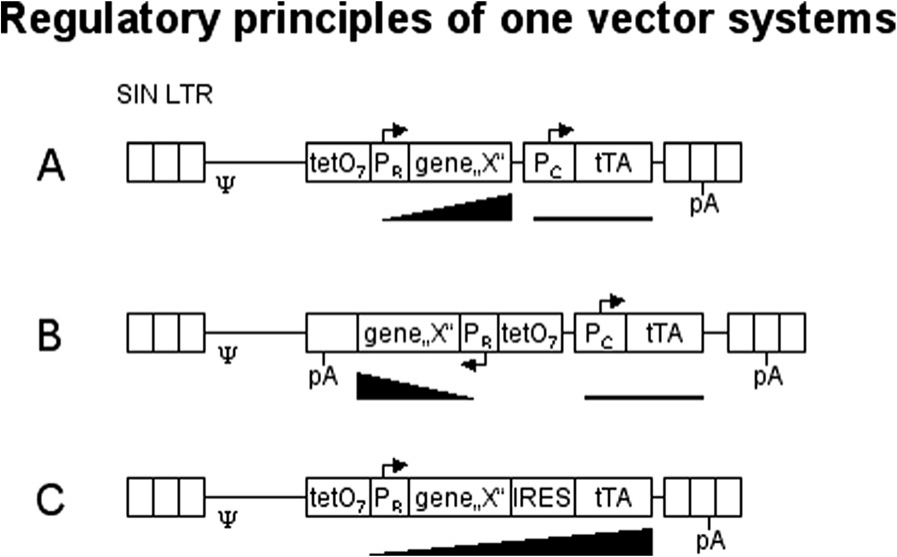
**Regulatory principles of retroviral “One-vector“ systems. (A)** Unidirectional provirus transferring a “self-contained“ One-vector system. The tet-responsive promoter (TetO_7_: tet-operator heptamer; P_R_: regulatable minimal Promoter) drives expression of the gene of interest (gene “X“). Its inducibility is indicated by the black triangle. A constitutive promoter (P_C_) drives expression of the tet-dependent transactivator (here exemplified by tTA) at a constant low level (black line). The unidirectional system utilizes the viral pA signal to terminate both transcripts and thereby generates overlapping transcripts. **(B)** The bidirectional system generates two distinct mRNAs, thus requires the insertion of an antisense orientated (−strand) pA-signal. **(C)** Unidirectional provirus transferring an “autoregulated“ One-vector system. A bicistronic unit couples the open reading frames of the gene of interest and the transactivator via an internal ribosomal entry site (IRES). The expression of both genes is driven by the inducible promoter (P_R_).

The most commonly applied gene regulation system is the tetracycline inducible gene expression (tet-) system, originally described by Gossen and Bujard [1]. It allows effector dose-dependent regulation and consists of two components, a tetracycline controlled transactivator (tTA) and a tet-responsive promoter (TRP) regulating the gene of interest. The transactivator binds with high affinity to the tetR-moiety of the TRP, a minimal promoter physically linked to the tet-operator sequence. Two transactivator variants have been developed, differing primarily in their response to the effector molecule tetracycline. In the Tet-off system, the tTA is released from its DNA binding site in the presence of doxycycline (Dox), a tetracycline derivative, thus abolishing gene expression. The opposite is true for the reverse transactivators rtTA2s-M2 and rtTA-3 [1-4] in the Tet-on system. Stable tet-controlled gene expression requires the transfer of both (r)TA and TRP into the target cell. Their step-wise integration/selection ensures independence of the constitutive transactivator expression unit from the TRP driven regulated gene expression, thereby enabling the selection of highly regulated clones. However, this strategy can not successfully be applied to systems where clonal selection is difficult or undesirable e.g. primary cells. To overcome this hurdle, so called “One-vector” systems were developed that allow for simultaneous integration of both components. These technologies have mostly been explored in the field of gene therapy, where primary cells are the major target. Almost all approaches were based on either retroviral or lentiviral vectors, since they allow for highly-efficient and stable integration of DNA into the host genome. Regarding the mode of transactivator expression, two systems have been applied. Transactivator expression is controlled by a constitutive promoter in self-contained vectors (Figure 1A) [5-15], while both transactivator and transgene expression is driven by the TRP in autoregulated vectors (Figure 1C) [10,16-20]. So far, for both vectors dose-dependent induction, as determined by either luciferase or GFP reporter gene expression, did not exceed 400-fold, with best regulatory properties being observed in clones rather than cell populations. While in self-contained vectors potential promoter crosstalk between constitutive promoter and TRP might be responsible for the observed low dynamic range [21], in autoregulated vectors basal expression of the inducible cassette is an essential requirement for initiation of the positive feedback loop. However, autoregulated vectors were generally favored when employing Tet-on systems, since low transactivator abundance during the ”off-state“ minimizes potential cytotoxicity [22,23] and immunogenicity [24-26]. Additional problems arise in “One-vector” systems, where transgene and transactivator reside on one viral backbone. Unidirectional expression of the two components can either be achieved by construction of bicistronic units (autoregulation) or by overlapping transcripts employing two promoters (self-contained). In both cases, transcription terminates at the polyadenylation (pA) signal located in the 3´-LTR, and expression levels were shown to be negatively affected [14,27]. In order to overcome this obstacle, bidirectional transfer vectors were constructed as illustrated in Figure 1B. Although proof of concept has been demonstrated for autoregulated bidirectional TRP [19], only moderate induction rates were achieved. Applying bidirectional lentiviral vectors of the self-contained type [28,29] resulted in a dynamic gene induction range of around 50-100-fold. Only one such approach has been reported for retroviral vectors [27], where application of improved TRPs resulted in an excellent dynamic range of more than 1000-fold.

In this study, we combined the key benefits of the self-contained and the autoregulated system with a bidirectional vector design. The two vectors explored in this study differed regarding their mode of transactivator expression. In the “self-contained” MOV-scT6 vector, M2 transactivator expression is under control of the human PGK promoter [27], while in the “autoregulated” MOV-scT6cA vector M2 transactivator expression is driven by the newly developed synthetic "cA" promoter, a weak constitutive but inducible minimal promoter. Selected cell populations were used to compare the regulatory properties of both vectors with respect to their effector dose–response and kinetics of activation.

## Results

### Design of the bidirectional vectors

**Figure 2:**
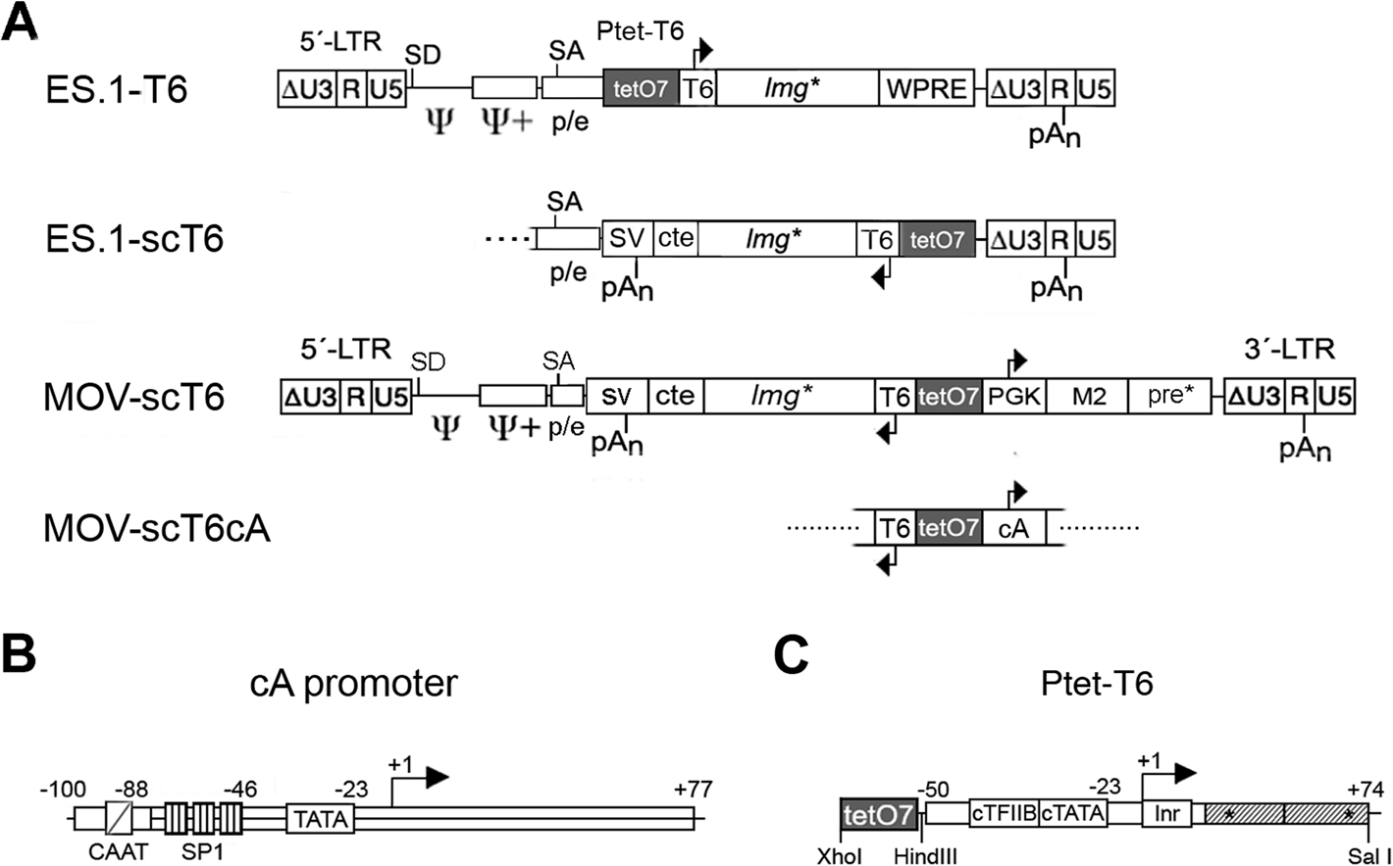
**Viral vectors and promoters. (A)** Provirus of the retroviral SIN-vector, ES.1 [32], with deleted viral enhancers (U3), contains enlarged packaging region (psi, psi+) and *pol/env* fragment harboring the native splice acceptor (SA). The tet-regulatory expression unit is inserted in sense orientation (+strand) followed by the woodchuck hepatitis virus posttranscriptional regulatory element (WPRE, [33]). The tet-responsive promoter, Ptet-T6, was functionally coupled to the dual reporter gene lmg*, for simultaneous determination of luciferase and GFP activities. ES.1-scT6 viral vector contains an inverted tet-regulatory expression unit (−strand), transcripts were terminated at the SV40 pA site that was fused to the constitutive transport element of SRV-1 (cte, [30]). The MOV backbone is identical with ES.1-scT6, but contained a sense (+strand) insertion of the PGK- or cA-promoter driven M2 transactivator [2]. The woodchuck hepatitis virus posttranscriptional regulatory element (pre*) differs in length from the ES.1 version. **(B)** Outline of the weak constitutive (“c“) but tet-inducible (“A“) cA-promoter. The CAAT box of MoMuLV was added to a HIV-1 derived minimal promoter −77/+77, containing 3 SP-1 sites and the TATA-box. Positions are numbered relative to the transcriptional start site. **(C)** The tet-responsive minimal promoter (Ptet) used in this study, Ptet-T6, consists of a synthetic minimal promoter with consensus TATA-box and TFIIB binding site, the CMV initiator element and a TYMV 5´-UTR assembled with a tet-operator heptamer with core-spacing of 36 nt [31]. Specific restriction sites are indicated.

As recently shown (Loew et al., 2010), introduction of the tet-responsive Ptet-T6 promoter (Figure 2C) into the ES.1 retroviral vector (ES.1-T6) resulted in an excellent dynamic range of reporter gene expression in transduced Hela-EM2 cells, constitutively expressing the doxycycline (Dox) responsive reverse M2-transactivator. Mo**M**uLV-based **O**ne-**V**ector systems (MOV) were constructed by insertion of a bidirectional expression cassette into the ES.1 backbone (Figure 2A). Transcripts initiated at Ptet-T6 were terminated at an antisense orientated SV40-pA signal (3´-5´, relative to the viral vector genome), fused to the constitutive transport element (cte) of simian retrovirus 1 [9,30]. To determine transgene expression levels in cell pools as well as at the single cell level, the dual reporter gene lmg*[31] was employed, enabling simultaneous determination of luciferase activity and eGFP fluorescence.

Two MOV-vectors were constructed, “self-contained” (MOV-scT6) and “autoregulated” (MOV-scT6cA), where M2-transactivator expression was either placed under control of the constitutive human phosphoglycerate kinase promoter (hPGK), or a newly designed tet-responsive “cA”-promoter (Figure 2B**,** see below). MOV vectors also contained a shortened version of the woodchuck hepatitis virus posttranscriptional regulatory element (pre*s; Additional file 1: Figure S2). M2 transcripts were terminated at the pA signal of the viral 3´-LTR.

### Properties of the regulatory unit within monocistronic vectors and the self-contained bidirectional vector

For a comparative analysis of the tet-responsive promoter, the TRP-unit was inverted within the monocistronic ES.1-T6 vector (excluding interference with the constitutive promoter (PGK)), thus resembling the orientation of the TRP-unit within the MOV-vector setting (Figure 2A, C). Following transduction and FACS-based enrichment of Hela-EM2 cells, activities were analyzed in the “on-/off-states”. While background expression remained fairly constant, the inducible activity of ES.1-scT6 was found to be decreased, resulting in an overall reduction in gene regulation by 60% (Table 1). Since the observed phenomenon can be explained by the absence of the pre*s element from the resulting transcript, Ptet-T6 was considered to function independent of the orientation. Subsequent insertion of the PGK-M2 expression unit into the ES.1-scT6 backbone resulted in the “self-contained” MOV-scT6 vector (Figure 2A). Determination of luciferase activity in transduced Hela cell populations indicated that both, background expression as well as inducible activity were negatively affected by the insertion, resulting in a reduction of the dynamic range (1300-fold) by about 70%, when compared to the parental ES.1-scT6 vector (4000-fold). While the presence of the constitutive promoter might directly account for the observed slight increase in background expression level, the decrease in gene induction levels might be explained by an insufficient concentration of M2-transactivator generated by the PGK-promoter.

**Table 1:** Expression level and regulatory potential of unidirectional and bidirectional vectors

	**On**	**Off**	**Induction**	
**Construct**	**(rlu/μg)**			**Cells**
	**(x 10^7^)**	**(x10^3^)**	**(x10^3^)**	
ES.1-Ptet-T6	4.1 ± 0.6	4.1 ± 0.1	9.8 ± 1.5	HeLa-EM2
ES.1-scPtet-T6	1.9 ± 0.2	4.7 ± 0.6	4.0 ± 0.8	HeLa-EM2
MOV-scT6	1.2 ± 0.01	9.2 ± 2.0	1.3 ± 0,3	HeLa

Luciferase activity (rlu/μg protein) was determined after enrichment of transduced Hela-EM2 cells (ES.1 vectors) constitutively providing the M2 transactivator, or Hela cells (MOV-vector) in the presence (“on“) or absence (“off“) of doxycycline (500 ng/ml). Induction was calculated from the activities determined in the on and off state. ± values reflect SEM. For each vector, two populations were established by one round of sorting (pool purity >90%) and were measured twice.

Although the dynamic range of gene regulation was shown to exceed previously published One-vector systems, further improvement was necessary to obtain full induction.

### Replacement of the constitutive PGK-promoter by an artificial inducible-promoter

To improve vector performance, we developed a minimal promoter designed to inhibit weak constitutive as well as inducible activity, thus introducing the autoregulated principle into bidirectional vectors. The newly designed cA promoter (Figure 2A, Additional file 2: Figure S1) consists of an HIV-1 minimal promoter, with low background activity in the context of a TRP [30] fused to the CAAT-box of the MoMuLV-LTR promoter. The latter was shown to be sufficient to provide residual activity of a minimal LTR [18]. This promoter was designed (i) to minimize crosstalk with the TRP, and (ii) to guarantee low basal levels of M2 transactivator during the “off-state”, while being sufficiently active to initiate the positive feedback loop. Replacing the PGK by the cA-promoter resulted in the generation of MOV-scT6cA vector (Figure 2A), which was considered to be autoregulated, providing a low constitutive activity for M2 transactivator expression.

**Figure 3:**
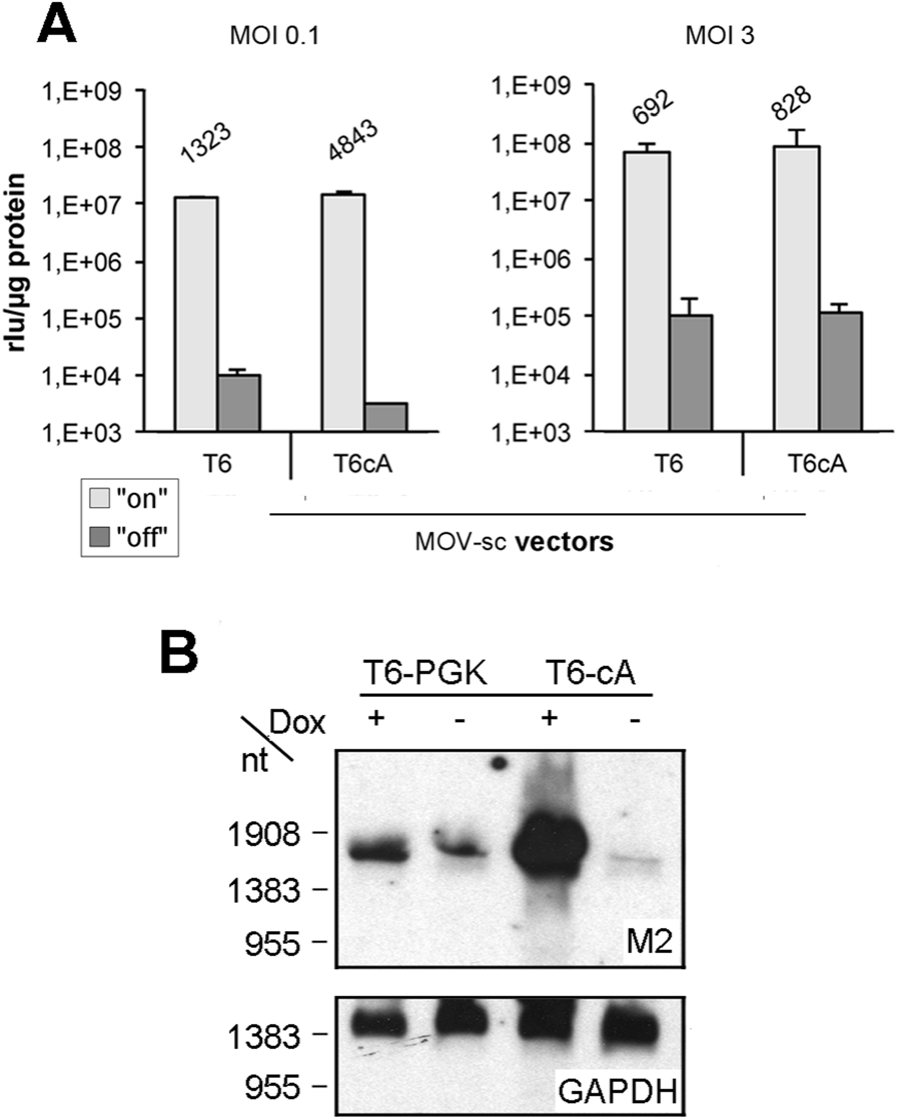
**Comparison of self-contained and autoregulated OneVector System. (A)** Determination of luciferase activity of Ht1080 cell populations transduced by either MOV-scT6 or MOV-scT6cA and enriched by FACS. The left panel shows the luciferase activity determined in the on/off state of the system after transduction with low MOI, the right panel after transduction with high MOI. Two independent populations were generated at the indicated condition and each was measured twice. Induction was calculated from the luciferase activities determined in the on/off state and given above the bars. **(B)** Northern blot analysis of representative Ht1080 cell populations transduced by either MOV-scT6 or MOVscT6cA. The blot was probed for the M2 transactivator or GAPDH.

Comparison of the self-contained vector MOV-scT6 and autoregulated vector MOV-scT6cA was performed in Ht1080 cell populations transduced at low MOI and enriched by FACS (Figure 3A, left). While cell populations derived from the autoregulated vector showed reduced background expression, the level of induction was maintained (1.3 and 1.4×10^7^ rlu/μg protein, respectively), resulting in a 3.7-fold increase in the dynamic range of gene regulation. Northern analysis (Figure 3B) of M2 steady state mRNA levels revealed reduced levels for the autoregulated vector under non-inducing conditions, while levels strongly increased upon induction. Interestingly, similar yet weaker effects were found for the self-contained vector (see below).

Increasing gene dosage (Figure 3A, right) strongly enhanced gene expression upon induction (up to 10^8^ rlu/μg), while the dynamic range of gene regulation was reduced. This phenomenon was observed in both vector systems. Based on luciferase data, a reduction in background activity could only be demonstrated for cell populations of the autoregulated vector, transduced at low MOI. This observation reveals the impact of the integration site, since under this condition variation due to position effect is pronounced.

Furthermore, severe effects on cell growth were observed for the autoregulated vector system, when cells were treated with high gene dosage, followed by induction (Additional file 3: Figure S3). This effect can most likely be attributed to high transactivator abundance and hence squelching.

### Autoregulation altered the mode of induced gene expression

As generally accepted, tet-controlled gene expression enables effector-dose dependent adjustment of transgene steady state levels. Therefore, dose–response experiments were performed to further characterize the two construction principles.

**Figure 4:**
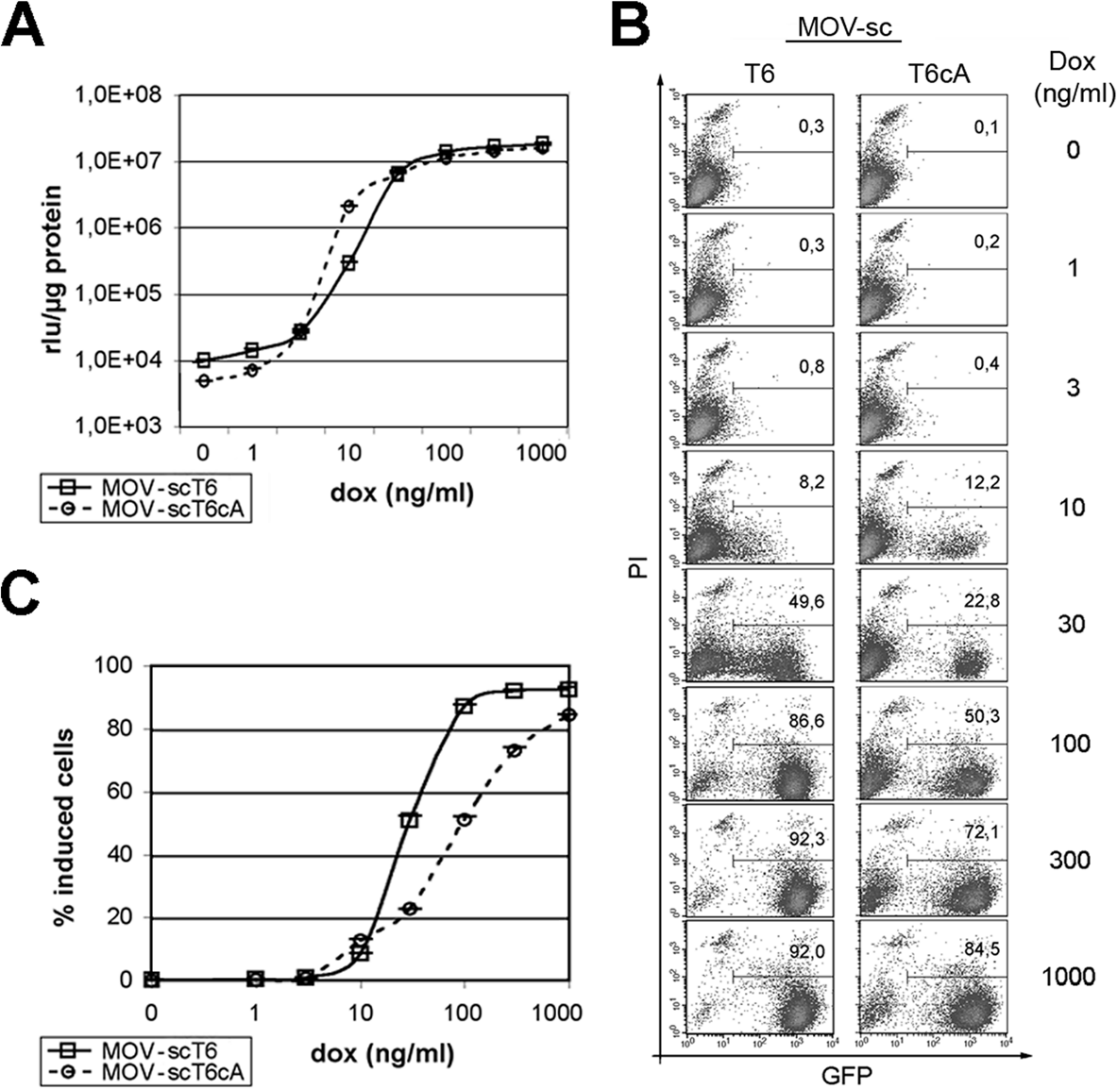
**Dose response kinetic. (A)** Luciferase activity of enriched Ht1080 cell populations transduced with MOV-scT6 or MOV-scT6cA vectors. Doxycycline concentrations were kept constant for four days by daily medium exchange. Values represent data from two independently generated populations. All measurements were accomplished in duplicate. **(B)** One representative population was used for a parallel determination of GFP fluorescence by flow cytometry. The percent GFP-positive cells (x-axis) are given within each blot measured against propidium iodide (PI, 1 μg/ml) stained dead cells (y-axis). It should be noted, that the purity of the enriched populations differed slightly. **(C)** Percent positive cells of the two independently generated populations. All measurements were accomplished in duplicate.

Ht1080 cell populations transduced by either MOV-scT6 or MOV-scT6cA vectors were cultivated in the “off-state”, following cell sorting for a minimum of 10 days. Cells were induced for 96 hours at the indicated Dox concentrations (Figure 4) to allow for adjustment of the steady state expression levels. Determination of luciferase activity (Figure 4A) revealed a similar induction response for both vectors, whereby full activation of the reporter gene expression was demonstrated at effector concentrations of around 300 ng Dox/ml. However, at low effector (Dox) concentrations, populations transduced by MOV-scT6cA displayed reduced background activity yet slightly increased induction rates, indicating an increased dynamic range for the autoregulated vector.

Further differences between the two vectors were revealed by FACS-based analysis of enriched Ht1080 cell populations (Figure 4B). As expected, transgene expression was found to be effector dose-dependent in cells transduced by the self-contained MOV-scT6 vector, with considerable intermediate levels at 10–100 ng Dox/ml. In contrast, full induction rates were observed at already lower effector concentrations for the autoregulated MOV-scT6cA vector and further increase in effector (Dox) concentration resulted only in increased numbers of induced cells (Figure 4C). Therefore, kinetics of the autoregulated vector rather resembled a threshold mode.

**Figure 5:**
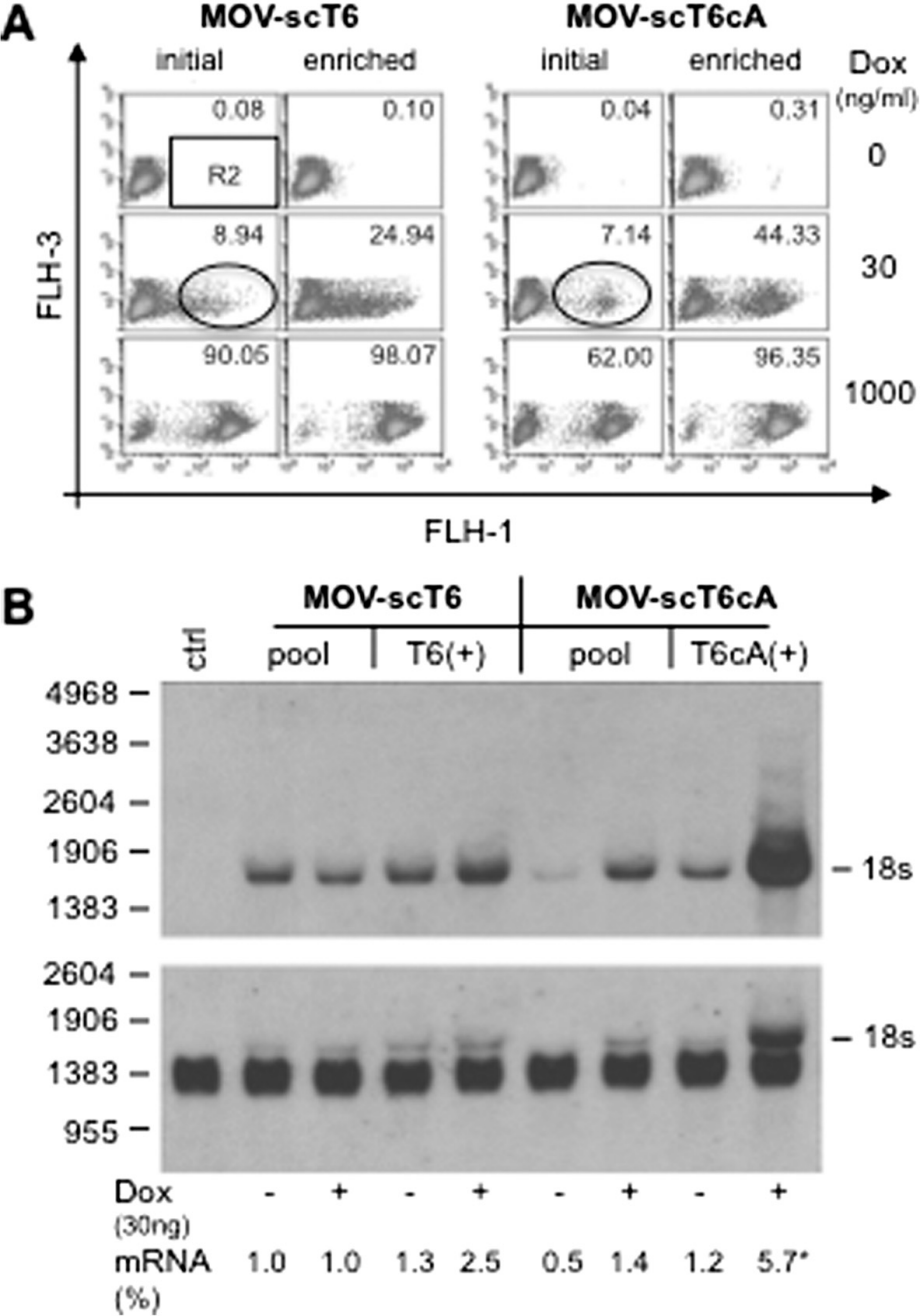
**Ht1080 populations transduced by either MOV-scT6 or MOV-scT6cA were enriched via FACS.** From both initial populations the fractions of cells inducible at 30 ng/ml Dox (circles) were enriched close to homogeneity. **(A)** Fluorescence based analysis of induction profiles of initial and enriched populations. The percentage of inducible cells (R2) at different Dox concentrations is inserted into the blots. **(B)** Northern blot analysis of initial (pool) and enriched (T6+ or T6cA+) populations. Total RNA was extracted from cells induced for 96 hrs with 30 ng/ml Dox and analysed after separation on 1.2% Agarose-MOPS-formaldehyde gel. Detection was performed with biotinylated probes against the M2-transactivator or GAPDH. Because of subsequent development of the blots, the M2 signals were partially visible in the blot probed with GAPDH. Densitometric analysis was done on appropriate developed blots (NIH 1.57 software), the relative values obtained for MOV-scT6 cells in the off-state (pool -Dox) was set to 1. The signal intensity of the T6cA+ could not be triggered into a linear range of signal intensity (*).

According to the law of mass action, the ability to display a threshold response should be dependent on the abundance of the M2 transactivator during the off-state (since it triggers the positive feed back loop) and therefore on the basal activity of the cA-promoter. For further clarification, sub-populations of the originally tested cell pools displaying high induction levels at low effector concentrations (30 ng/ml Dox) were enriched (>95%, Figure 5A). Total RNA was prepared from the populations in the on- and off-states and analyzed by Northern blot for steady state levels of M2-mRNA. Comparison of the initial and enriched sub-populations for the self-contained MOV-scT6 vector revealed only minor differences (1 vs. 1.3) during the off-state. However, induction at 30 ng/ml Dox led to a 2.5-fold increase in M2-mRNA in the enriched population. Contrary to what we observed for the self-contained vector, analysis of the autoregulated MOV-scT6cA vector showed that M2-mRNA levels in the off-state were approximately doubled (0.5 vs. 1.2) in the enriched population and strongly increased upon induction at low Dox concentration (Figure 5B). Yet, only a subset of cells of the enriched populations (25% for MOV-scT6 and 44% for MOV-scT6cA, respectively) exhibited induction at 30 ng/ml Dox (Figure 5A), while full induction could be achieved at maximum effector rates, 1000 ng/ml Dox (>95%). We therefore assumed, that in the remaining cells at low effector concentrations M2 transactivator levels might not be sufficient i) to saturate the tet-operators of the TRP or with respect to the autoregulated vector ii) to trigger the positive feedback loop.

**Figure 6:**
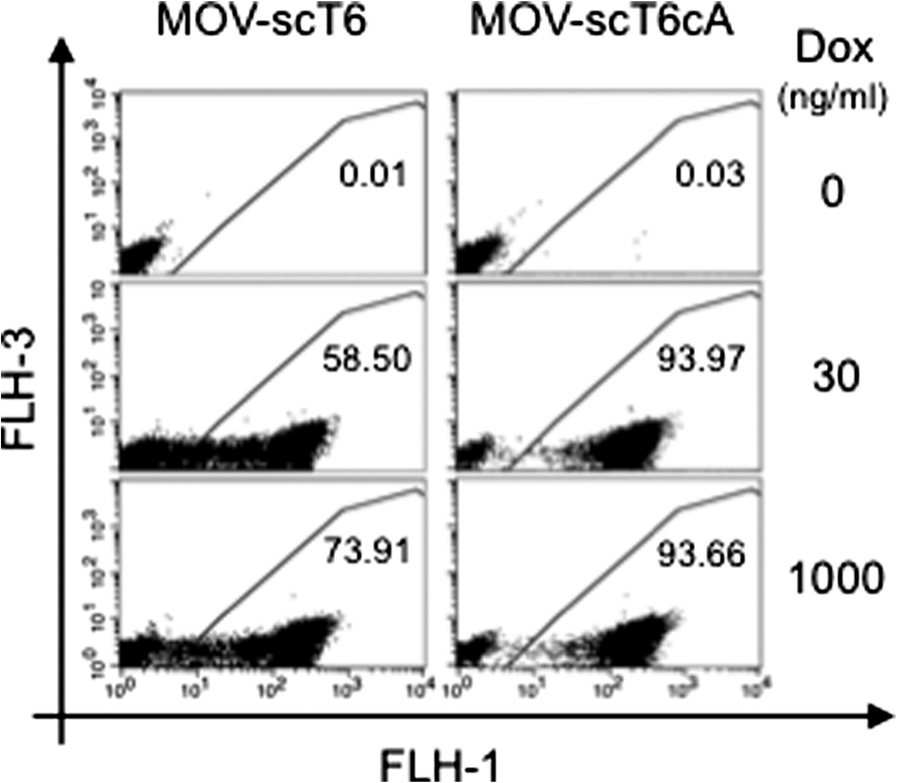
**Hela-EM2 cells transduced and enriched by FACS.** Cells were induced for 96 hrs with the indicated Dox concentrations. The percent of GFP positive cells was inserted into the blots.

Increasing the overall cellular abundance of M2 transactivator might be an approach to overcome this obstacle. To test this hypothesis, Hela-EM2 cells, which provide background levels of M2-transactivator via the EF1-promoter, were transduced with either MOV-scT6 or MOV-scT6cA. Hela-EM2 pools were generated at low MOI and further enriched by one round of FACS. All cells transduced by the autoregulated vector MOV-scT6cA showed full induction at 30 ng/ml Dox (Figure 6). Surprisingly, a less pronounced effect could also be demonstrated for the self-contained vector.

Taken together, tet-regulated transgene expression was found to resemble a threshold mode in the autoregulated system. Following the law of mass action, full induction rates depended on the concentrations of M2-transactivator and its ligand (Dox), respectively. Variations observed at single cell level indicated insufficient M2-transactivator levels for a subset of the transduced cells. Since this could be overcome in systems were transactivator was provided from an independent locus, basal activity of the cA-promoter rather than of the TRP had been affected at the integration site.

### Induction kinetics of self-contained and autoregulated vectors

**Figure 7:**
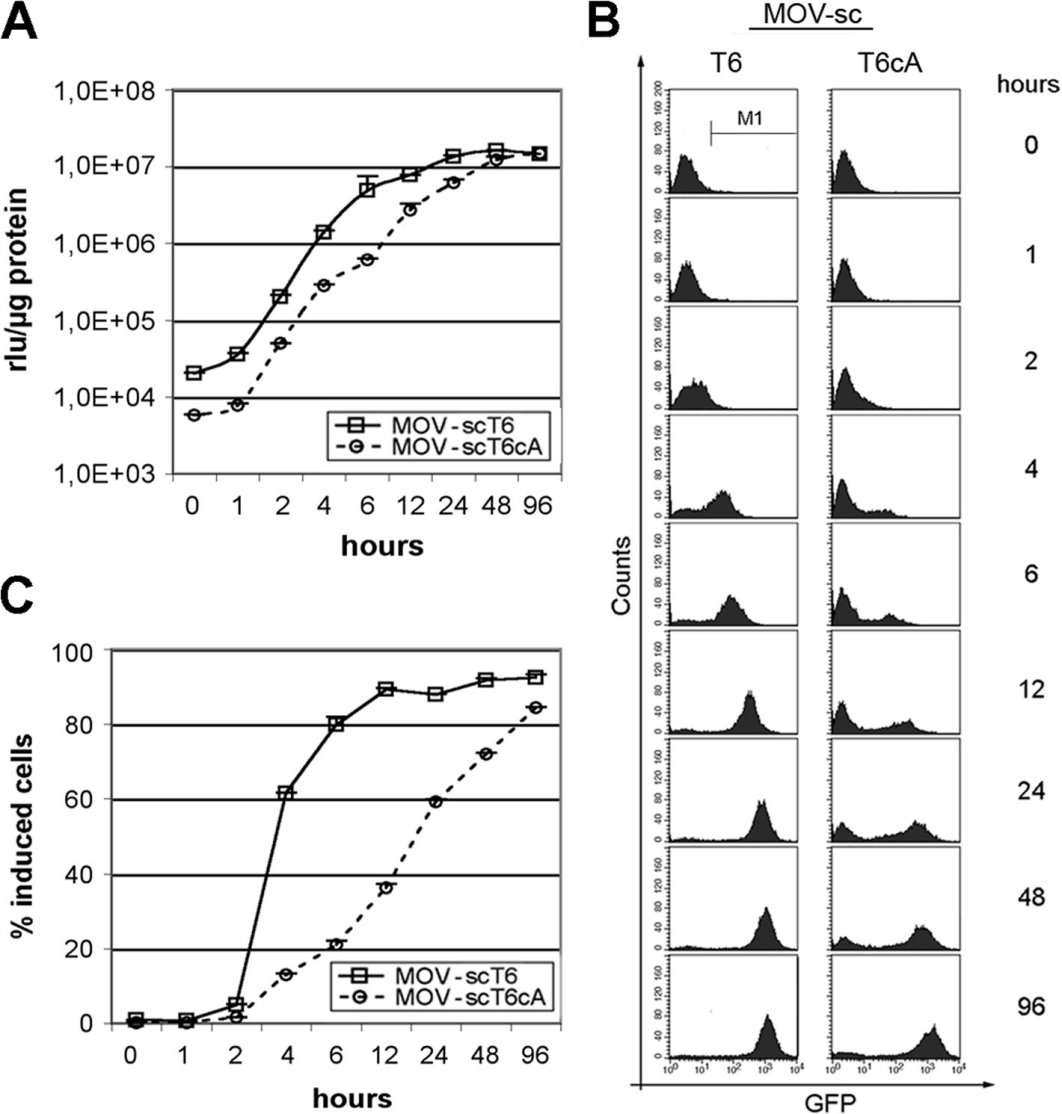
**Induction kinetics of MOV-scT6 and MOV-scT6cA. (A)** Luciferase activity of enriched Ht1080 cell populations transduced with MOV-T6sc or MOV-scT6cA vectors. Doxycycline concentration (1000 ng/ml) was kept constant during the experiment by daily medium exchange. Values represent data from two independently generated populations. All measurements were accomplished in duplicate. **(B)** One representative population is shown for a parallel determination of GFP fluorescence in FACS. The M1-region was used for the determination of the percentage of GFP positive cells. **(C)** Induced cells (reaching M1-region in “B“). Mean values of two independently generated populations. All measurements were done as duplicates.

Since only subsets of cells transduced by the autoregulated vector had the potential to become fully activated at low Dox concentrations, especially after enrichment (Figure 6), populations generated with the self-contained vector were thought to display a different induction kinetic. Transduced Ht1080 populations were cultured in saturating Dox concentrations (1000 ng/ml) for different time periods and Luciferase activity was analyzed. As expected, populations of the self-contained MOV-scT6 vector displayed faster induction kinetics, before they finally reached a steady state level (Figure 7A). This finding was further supported by GFP fluorescence analysis at single cell level (Figure 7B). MOV-scT6 transduced cells migrated as total population starting about 2 hours following induction, reaching a maximum of GFP accumulation within 24–48 hours. In contrast, only a subset of MOV-scT6cA transduced cells showed a fast response upon induction, while the majority of cells remained at the background level, indicating temporal control of the positive feedback loop.

This important difference is further illustrated in Figure 7C. While about 60% of the cells transduced by the self-contained MOV-scT6 vector showed clear induction after about 4 hours, only about 10% of the population transduced by the autoregulated MOV-scT6cA vector displayed a fast response. During further induction, the percentage of induced cells increased only slowly compared to the rapid activation of all cells transduced by the self-contained MOV-scT6 vector, suggesting involvement of particular cellular events, which influence the chromosomal environment and thereby the activity of the TRP/cA promoter.

## Discussion

Since the mid 90‘s, numerous studies have explored strategies for simultaneous (and reliable) transfer of both tet-system components into target cells. However, achieving tight control in “One-vector systems” has remained a challenge, as the dynamic range of gene expression was found to be hampered by high background and/or low transgene expression. In this study, we report on the design of a new MoMuLV-based One-vector system, with promising features. Firstly, open reading frames of the two components were expressed bidirectionally. Overlapping transcripts can thus be avoided, as these might reduce expression levels and negatively effect the dynamic range of tet-regulated gene expression [20,27,29]. Secondly, expression of the M2-transactivator was driven by the newly designed “cA” promoter, which exhibited weak basal as well as inducible activity. Results obtained from Ht1080 cell populations transduced with either the newly designed autoregulated vector, MOV-scT6cA, or the self-contained vector, MOV-scT6, demonstrate the superiority of the developed One-vector system (Figure 3). While both vectors showed high inducible expression, based on luciferase activity (bulk assay), the dynamic range of gene regulation in the autoregulated MOV-scT6cA vector was found to be increased by 3.7-fold as compared with the self-contained MOV-scT6 vector (4.8×10^3^ vs. 1.3×10^3^-fold). This improvement was largely due to the reduced background activity in the autoregulated MOV-scT6cA vector. Our results further suggest that promoter interference [21] between the tet-responsive Ptet-T6 and cA-promoter was reduced compared to the combination of Ptet-T6 and PGK-promoter and that a selection for integration sites promoting basal activity of the TRP/cA-promoter did not occur. These observations are in accordance with the findings of Lindemann and co-workers [18], who reported best results for an autoregulated MoMuLV-based system with respect to expression levels and regulatory properties *in vitro* and *in vivo*, when transactivator expression was driven by an enhancer-deleted LTR. Functionality of the cA-promoter design was further demonstrated by analysis of the M2-mRNA steady state level in the absence of Dox, revealing a 50% reduction compared to the PGK-promoter (Figure 5B). Infection of cell populations at increasing MOIs led to enhanced expression levels of the dual reporter gene lmg*, demonstrating an increase in gene dosage. However, at high MOI (≥1), cell populations transduced by MOV-scT6cA displayed strong growth retardation under inducing conditions (Additional file 3: Figure S3), suggesting massive accumulation of M2-transactivator to levels that caused squelching [23,34,35]. The moderate growth retardation observed in cells transduced by MOV-scT6 might be explained by exhaustion of other essential cell components, e.g. amino acids or nucleotides, since here expression levels of the dual reporter gene lmg* went into extremes (>4×10^7^ rlu/μg protein).

As expected, the dose–response analysis of the two vector types, self-contained (MOV-scT6) and autoregulated (MOV-scT6cA), revealed a significant difference in their response mechanism (Figures 4 and 5). While the self-contained vector exhibited a more graded, Dox-dependent induction of gene expression [36,37], a threshold mode was observed for the autoregulated vector. This important difference was only detected at the single cell level, as demonstrated in cell based analysis of eGFP fluorescence of the dual reporter gene lmg*, since it was masked in luciferase analysis of bulk cultures.

Markusic and co-workers obtained similar results [10] by direct comparison of a self-contained and an autoregulated unidirectional lentiviral vector. In their study, populations transduced by the autoregulated vector displayed a nearly full induction of gene expression at yet intermediate effector (Dox) concentrations and an increase in positive cells at higher Dox concentration (Figure 5 in their paper). From the combined results it may be concluded that the threshold response was due to the autoregulated mode for transactivator expression. Further observations support the hypothesis that basal transactivator abundance might be the limiting factor: i) a sub population, enriched for its ability to achieve full induction levels at 30 ng/ml Dox, displayed an increased steady state level of M2-mRNA already before induction (Figure 5B), and (ii) Hela-EM2 cells, which provide a basal abundance of M2 transactivator, showed a threshold response of the total cell population at 30 ng/ml Dox, when transduced by the autoregulated MOV-scT6cA vector.

From these observations, a model following the law of mass action can be derived, with activation of transgene expression being proportional to the product of the concentrations of M2-transactivator and its effector Dox. Thus, full activation of the TRP-driven transgene could be achieved at low M2-transactivator levels, given that effector concentration remained at optimum level (Figures 4, 5; 1000 ng/ml Dox), or, vice versa, at high levels of M2-transactivator at otherwise limiting Dox concentrations (Figures 5, 6; 30 ng/ml Dox).

Our data further suggest that the basal activity of the cA-promoter is dependent upon the integration sites. Only loci that favoured the start of the autoregulated circuit were able to induce the threshold response of the Tet-system at low Dox concentrations. The accessibility of the TRP at the chromosomal integration site seems to be of minor importance for the conversion of the graded to a threshold response.

## Conclusions

In summary, our results demonstrated the advantageous properties of the autoregulatory compared to the self-contained principle for M2-transactivator expression, when using retroviral vectors with a bidirectional design, combined with the inducible cA-promoter. However, limitations occur when high vector dosages are applied. In particular, the observed on/off switch may have significant advantages, especially considering that full activation was achieved at suboptimal Dox concentrations and thus might help to overcome induction problems related to tissue-specific barriers for effector penetration. However, graded induction of gene expression is not possible with the autoregulated cA promoter and thus excludes this promoter design from experiments where an adjustable mode of transgene expression is mandatory. Moreover, the dependence of induced gene expression on the cellular abundance of the transactivator provides important evidence to help explain the large difference of effector concentrations reported to fully activate TRPs in various cell systems.

## Methods

### Cell culture

293T (ATCC # CRL-11268), Hela-EM2 [38] and Ht1080 cells were cultured in Dulbecco´s modified Eagles medium (DMEM, Invitrogen) supplemented with 10%, heat inactivated fetal bovine serum (FBS, PAA) at 5% CO_2_ and 37°C. Cultures were split at 70-80% confluency. Following a washing step with PBS and incubation for 3–5 min in the presence of PBS/EDTA (0,8 mM), cells were harvested and either transferred into fresh medium or used in subsequent analysis.

### Transient vector production and titration

Transient production of viral vectors was carried out by lipofection with the TransIt293 reagent (Mirus, CA) as recommended by the supplier. About 1.5×10^6^ 293T cells were transferred to 60 mm dishes the day before transfection. A total amount of 15 μg plasmid DNA was transfected containing 5 μg pHIT60 (gag/pol expression plasmid; [39]), 5 μg pczVSV-G (VSV-G envelope expression plasmid [40]) and 5 μg of the transfer vector. 16–18 hours after transfection the medium was replaced by 3 ml DMEM-medium, supplemented with 5 mM Na-butyrate, which was exchanged for DMEM-medium without Na-butyrate after additional 6–8 hours. 16–18 hours following medium exchange the supernatant was harvested, filtrated (0,45 μm, Nunc), supplemented with polybrene (5 μg/ml, SIGMA), aliquoted and stored at −80°C for later use.

All titrations were performed on Ht1080 cells using serial dilutions of the obtained supernatants (5-10-20-40-80-160-fold, respectively). Briefly, 2×10^5^ cells were transferred to a 6well plate the day before infection. 24 hours later medium was replaced by 1 ml of fresh culture medium supplemented with polybrene (5 μg/ml) and premixed with supernatant. After about 18–20 hours medium was renewed and cells were cultivated under induced conditions (Dox 1000 ng/ml). Fluorescence activated cell sorting (FACS) or otherwise analysis of cell populations were performed on day 6 (about 96 hours post induction). For calculation of viral titers the number of GFP positive cells (about 4×10^5^ cells × % GFPpos/100) was determined, a correction factor of 2 was applied to account for cell division during infection. In general, titers in the range of 1-3×10^6^ IP/ml could be obtained.

### Establishing transduced cell populations

About 4×10^5^ cells (Hela-M2) were infected (always in the absence of Dox) on 6well plates with serial dilutions of the transiently produced vectors and induced after the first split for four to five days at 1000 ng/ml Dox. Appropriate infected populations (1-3% positive cells) were used for the enrichment by one round of FACS. These conditions ensured, that mostly single copy integrates of the vectors were generated. In general, the established individual populations were adjusted to present >15.000 independent clones.

### Determination of luciferase activity

Purified transduced cell populations had to be cultivated in the “off-state” for a period of least 10 days, due to the prolonged half life of luciferase in the fusion protein lmg* and the high expression level of the tet-units. Induction experiments were started by splitting 0.5-1×10^5^ cells into cell culture medium with or without Dox (500 ng/ml). After 96 (72) hours incubation cells were harvested with PBS/EDTA and GFP fluorescence and luciferase activity were analyzed simultaneously. 0.5-2 μl of bulk cell lysate were used for analysis of luciferase activity by luminescence detection (Lumat, Berthold, Germany), essentially as described earlier [41]. Protein concentration was determined according to the method of Bradford [42] and specific luciferase activity was calculated.

In general, treatment of cells was similar in dose response experiments, except for a daily medium exchange. This was applied in order to counteract the potential degradation of Dox, which may affect the level of induction especially at low concentrations. Medium was supplemented with the indicated Dox-concentrations.

Experiments on induction kinetics required transfer of individual cell numbers, thus, allowing the harvest of a sufficient amount of cells for short term cultures, and avoiding overgrowth of the cells used for prolonged cultivation. In general, cells for short term analysis (e.g. 0.5 hours of induction) were splitted to high density (5×10^5^ cells/6well), while cells for the 24/48/72/96 hours induction were transferred at about 4-2-1 or 0.5 × 10^5^ cells/6well.

### Northern analysis of total RNA

For RNA analysis the enriched populations were grown on 9cm dishes either in the absence or presence of Dox. After 96 hours the cells were harvested and total RNA was extracted by the acidic phenol method [43]. Northern analysis was performed as described earlier [44]. Detection was carried out with avidin conjugated alkaline phosphatase (Molecular Probes) and CDP-Star (Tropix) as substrate for chemiluminescent detection. Rat GAPDH cDNA served as an internal mRNA standard. All probes used were biotin-labeled during PCR-synthesis. Detection of the mRNA steady states was achieved by exposure to X-Ray film (Kodak Bio-Max light, Sigma). Sizes of the RNA marker (Promega) are indicated in the figures. The following oligonucleotides were used for probe synthesis: sense 5´- TTACAGATGCACATATCGAGG, antisense: 5´-CCTCTGGATCTACTGGGTTA (rat GAPDH) and sense 5´- tctagactggacaagagc, antisense: 5’- ccgccgctttcgcactt (rtTA2s-M2). Densitometric analysis of appropriately exposed films was performed by use of NIH 1.57 software.

### Plasmid constructs

The retroviral SIN-vector “pES.1” used for the transfer of the tet-response units had been described earlier [31].

The inducible expression cassette consisted of a tet-operator heptamer, the Ptet-T6 TRP, the dual reporter gene lmg* and a modified (see below) posttranscriptional regulatory element of the woodchuck hepatitis virus (WPRE, [45]). While the transcription of the ES.1-T6 vector was terminated at the pA-signal of the viral 3-LTR, the ES.1-T6sc transcripts were terminated at the antisense orientated SV40_(late)_ polyadenylation signal fused to the constitutive transport element (cte) of SRV-1 [32,46]. The tet-responsive promoter as all other components was subcloned into pBluescript SKII+ plasmid backbone (Stratagene, CA) by standard techniques [47] and sequenced (Eurofins, Germany).

The WPRE element, which already contained mutations of “atg´s” of the original element, was newly synthesized by PCR, using the SIN11 vector [33] as template. Sequence alignment to the WPRE used in the lentiviral vectors of the Naldini Lab [9] showed a 400 bp homologous stretch. This sequence, common to both WPRE elements, was PCR amplified and used for generation of the constructs (Additional file 1: Figure S2).

The cA-promoter was PCR amplified using the S2f-clHCg [30] as template. The CAAT-box of MoMuLV was introduced upstream of the SP-1 sites by amplification with the particular sense oligo. The full sequence is given in Additional file 2: Figure S1.

## Competing interests

The authors declare that they have no competing interests.

## Authors’ contributions

HN, HK and LR performed and analyzed the data and wrote the manuscript. All authors read and approved the final manuscript.

## Supplementary Material

Additional file 1 **Figure S2.** Alignment of the WPRE element used in the lentiviral pRRL.SIN. vector ([15], N, upper sequence), and the WPRE* element as used in the SIN11 retroviral vector ([33] B, lower sequence). Mutations introduced to eliminate the “atg´s“ are boxed. The WPRE*-short fragment (pre*s) used throughout this work is underlined. Click here for file

Additional file 2 **Figure S1.** cA-promoter. Complete sequence of the artificial promoter is shown. 5´ and 3´ cloning sites are underlined. The MoMuLV sequence (*italic*) containing the CAAT-Box element was fused via PCR to the HIV-1 LTR fragment containing three SP1-sites (bold) and the TATA-box (underlined)*. * Click here for file

Additional file 3 **Figure S3.** Induced squelching at high multiplicity of infection (MOI). The increased steady state levels of M2 transactivator under inducing conditions (Figure 2C) implied, that especially for the auto-regulatory circuit the transactivator might accumulate to levels that were not tolerated by the cells and thus provoke collateral damage by squelching. The most consistent side effect related to squelching is a reduced growth capacity of the cells [23] and at later stages also a reduced overall capacity for gene induction, both most likely resulting from titrating out essential factors for the basal transcriptional machinery [48]. In order to verify this, we determined the luciferase activity as well as growth characteristics of cells transduced at low, intermediate and high multiplicity of infection (MOI 0.1, 1 and 3). It should be noted, that the populations generated at MOI 0.1 (generating 1-3% positive cells) were enriched by one round of FACS sorting, while MOI 1 and MOI 3 populations were measured without any enrichment. The results of the experiments (after 4 days of induction with 1000ng Dox/ml) indicated that the luciferase activity in the on- and the off-state correlated with the MOI in the transduced populations, although much less positive cells contributed to the luciferase activity, as was determined in FACS. Thus, increased gene transfer was established for both vectors resulting in a decreased dynamic range of gene regulation (~1000-fold induction) at MOI 3. The populations established with the self-contained MOV-scT6 vector displayed only a moderate decrease of cell growth, while growth of the populations established with auto-regulated MOV-scT6cA was strongly affected upon induction of gene expression. While growth of populations containing mostly a single copy integrate of the vector (MOI ≤0.1) was not decreased, an increased gene dosage lead to strong growth retardation after induction. Proposing that a higher gene dosage will lead to increased concentration of transactivator, this indeed may be a direct effect of squelching. The observation (not shown) that a prolonged induction was able to recover growth capacity by further reducing the proportion of positive cells in those populations supported this assumption as the residual, transgene negative cells started to overgrow the transgene positive cells. Click here for file
