# Supplementary material for: Graded or threshold response of the tet-controlled gene expression: all depends on the concentration of the transactivator
Source: BMC Biotechnol. 2013 Jan 22;13:5. doi: 10.1186/1472-6750-13-5 (PMC3556329; doi:10.1186/1472-6750-13-5)
Supplement: Additional file 1 — Figure S2. Alignment of the WPRE element used in the lentiviral pRRL.SIN. vector ([15], N, upper sequence), and the WPRE* element as used in the SIN11 retroviral vector ([33] B, lower sequence). Mutations introduced to eliminate the “atg´s“ are boxed. The WPRE*-short fragment (pre*s) used throughout this work is underlined. [file 1472-6750-13-5-S1.docx]

N --------------------------------------------------------------------------------

B ------GAGCATCTTACCGCCATTTATtCCCATATTTGTTCTGTTTTTCTTGATTTGGGTATACATTTAAATGTTAATAA

N --------------------------------------------------------------------------------

B AACAAAATGGTGGGGCAATCATTTACATTTTTAGGGATATGTAATTACTAGTTCAGGTGTATTGCCACAAGACAAACATG

N --------------------------------------aatcaacctctggattacaaaatttgtgaaagattgactggt

|||||||||||||||||||||||||||||||||||||||| |

B TTAAGAaactttcccgttatttacgctctgttcctgttAATCAACCTCTGGATTACAAAATTTGTGAAAGATTGACTGAT

N attcttaactatgttgctccttttacgctatgtggatacgctgctttaatgcctttgtatcatgctattgcttcccgtat

||||||||||||||||||||||||||||| |||||||| ||||||||||||||| ||||||||||||||||||||||||

B ATTCTTAACTATGTTGCTCCTTTTACGCTGTGTGGATATGCTGCTTTAATGCCTCTGTATCATGCTATTGCTTCCCGTAC

N ggctttcattttctcctccttgtataaatcctggttgctgtctctttatgaggagttgtggcccgttgtcaggcaacgtg

||||||| |||||||||||||||||||||||||||||||||||||||||||||||||||||||||||||| |||||||||

B GGCTTTCGTTTTCTCCTCCTTGTATAAATCCTGGTTGCTGTCTCTTTATGAGGAGTTGTGGCCCGTTGTCCGTCAACGTG

N gcgtggtgtgcactgtgtttgctgacgcaacccccactggttggggcattgccaccacctgtcagctcctttccgggact

|||||||||| |||||||||||||||||||||||||||| ||||||||||||||||||||||| |||||||| |||||||

B GCGTGGTGTGCTCTGTGTTTGCTGACGCAACCCCCACTGGCTGGGGCATTGCCACCACCTGTCAACTCCTTTCTGGGACT

N ttcgctttccccctccctattgccacggcggaactcatcgccgcctgccttgcccgctgctggacaggggctcggctgtt

||||||||||||||||| || |||||||| |||||||||||||||||||||||||||||||||||||||||| || || |

B TTCGCTTTCCCCCTCCCGATCGCCACGGCAGAACTCATCGCCGCCTGCCTTGCCCGCTGCTGGACAGGGGCTAGGTTGCT

N gggcactgacaattccgtggtgttgtcggggaagctgacgtcctttccatggctgctcgcctgtgttgccacctggattc

||||||||| |||||||||||||||||||||||||||||||||||||

B GGGCACTGATAATTCCGTGGTGTTGTCGGGGAAGCTGACGTCCTTTC=================================

N tgcgcgggacgtccttctgctacgtcccttcggccctcaatccagcggaccttccttcccgcggcctgctgccggctctg

B ================================================================================

N cggcctcttccgcgtcttcgccttcgccctcagacgagtcggatctccctttgggccgcctccccgcctgt

B =======================================================================

Supplementary Figure S2: Alignment of the WPRE element used in the lentiviral pRRL.SIN. vector ([15], N, upper sequence), and the WPRE* element as used in the SIN11 retroviral vector ([32] B, lower sequence). Mutations introduced to eliminate the „atg´s“ are boxed. The WPRE*-short fragment (pre*s) used throughout this work is underlined.
