# Supplementary material for: Graded or threshold response of the tet-controlled gene expression: all depends on the concentration of the transactivator
Source: BMC Biotechnol. 2013 Jan 22;13:5. doi: 10.1186/1472-6750-13-5 (PMC3556329; doi:10.1186/1472-6750-13-5)
Supplement: Additional file 2 — Figure S1. cA-promoter. Complete sequence of the artificial promoter is shown. 5´ and 3´ cloning sites are underlined. The MoMuLV sequence (italic) containing the CAAT-Box element was fused via PCR to the HIV-1 LTR fragment containing three SP1-sites (bold) and the TATA-box (underlined). [file 1472-6750-13-5-S2.docx]

**Supplementary Data**

XhoI

ctcgag*AACTAAccaatcAGTTC*caagg**GAGGCGTGGC**c**TGGGCGGGAC**t**GGGGAGTGGC**gagccctcagatgctgcatataagcagctgctttttgcctgtacTgggtctctctggttagaccagatttgagcctgggagctctctggctaactagggaacccactgcttaagcctcaataGAATTC

EcoRI

Supplementary Figure S1: cA-promoter. Complete sequence of the artificial promoter is shown. 5´ and 3´ cloning sites are underlined. The MoMuLV sequence (*italic*) containing the CAAT-Box element was fused via PCR to the HIV-1 LTR fragment containing three SP1-sites (bold) and the TATA-box (underlined)*.*
