# Supplementary material for: Graded or threshold response of the tet-controlled gene expression: all depends on the concentration of the transactivator
Source: BMC Biotechnol. 2013 Jan 22;13:5. doi: 10.1186/1472-6750-13-5 (PMC3556329; doi:10.1186/1472-6750-13-5)
Supplement: Additional file 3 — Figure S3. Induced squelching at high multiplicity of infection (MOI). The increased steady state levels of M2 transactivator under inducing conditions (Figure 2C) implied, that especially for the auto-regulatory circuit the transactivator might accumulate to levels that were not tolerated by the cells and thus provoke collateral damage by squelching. The most consistent side effect related to squelching is a reduced growth capacity of the cells [23] and at later stages also a reduced overall capacity for gene induction, both most likely resulting from titrating out essential factors for the basal transcriptional machinery [48]. In order to verify this, we determined the luciferase activity as well as growth characteristics of cells transduced at low, intermediate and high multiplicity of infection (MOI 0.1, 1 and 3). It should be noted, that the populations generated at MOI 0.1 (generating 1-3% positive cells) were enriched by one round of FACS sorting, while MOI 1 and MOI 3 populations were measured without any enrichment. The results of the experiments (after 4 days of induction with 1000ng Dox/ml) indicated that the luciferase activity in the on- and the off-state correlated with the MOI in the transduced populations, although much less positive cells contributed to the luciferase activity, as was determined in FACS. Thus, increased gene transfer was established for both vectors resulting in a decreased dynamic range of gene regulation (~1000-fold induction) at MOI 3. The populations established with the self-contained MOV-scT6 vector displayed only a moderate decrease of cell growth, while growth of the populations established with auto-regulated MOV-scT6cA was strongly affected upon induction of gene expression. While growth of populations containing mostly a single copy integrate of the vector (MOI ≤0.1) was not decreased, an increased gene dosage lead to strong growth retardation after induction. Proposing that a higher gene dosage will lead to increased con [file 1472-6750-13-5-S3.docx]

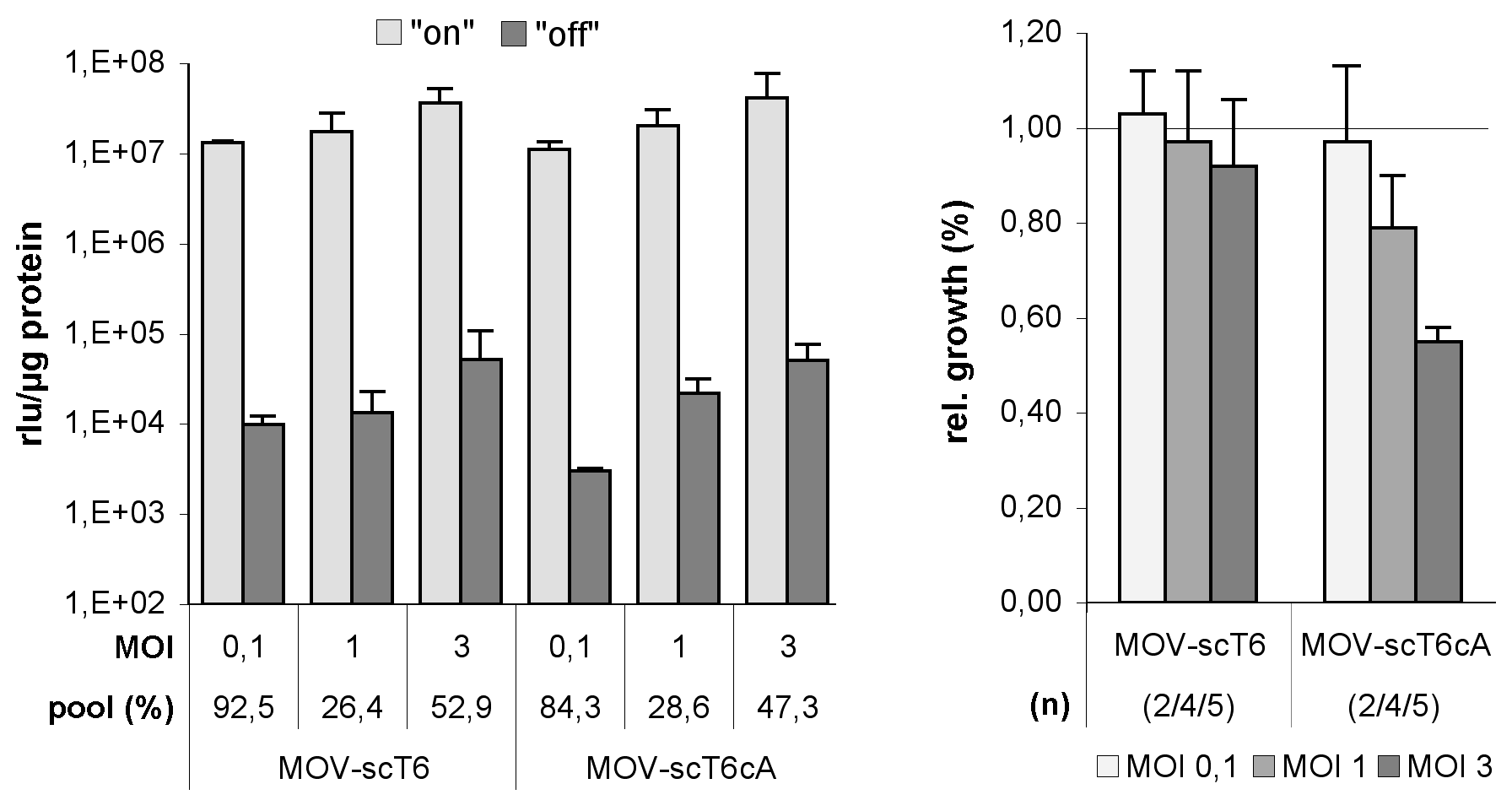


Supplementary Figure S3: Induced squelching at high multiplicity of infection (MOI). The increased steady state levels of M2 transactivator under inducing conditions (Figure 2C) implied, that especially for the auto-regulatory circuit the transactivator might accumulate to levels that were not tolerated by the cells and thus provoke collateral damage by squelching. The most consistent side effect related to squelching is a reduced growth capacity of the cells [23] and at later stages also a reduced overall capacity for gene induction, both most likely resulting from titrating out essential factors for the basal transcriptional machinery ^44^; [48]. In order to verify this, we determined the luciferase activity as well as growth characteristics of cells transduced at low, intermediate and high multiplicity of infection (MOI 0.1, 1 and 3). It should be noted, that the populations generated at MOI 0.1 (generating 1-3% positive cells) were enriched by one round of FACS sorting, while MOI 1 and MOI 3 populations were measured without any enrichment. The results of the experiments (after 4 days of induction with 1000ng Dox/ml) indicated that the luciferase activity in the on- and the off-state correlated with the MOI in the transduced populations, although much less positive cells contributed to the luciferase activity, as was determined in FACS. Thus, increased gene transfer was established for both vectors resulting in a decreased dynamic range of gene regulation (~1000-fold induction) at MOI 3.

The populations established with the self-contained MOV-scT6 vector displayed only a moderate decrease of cell growth, while growth of the populations established with auto-regulated MOV-scT6cA was strongly affected upon induction of gene expression. While growth of populations containing mostly a single copy integrate of the vector (MOI ≤0.1) was not decreased, an increased gene dosage lead to strong growth retardation after induction. Proposing that a higher gene dosage will lead to increased concentration of transactivator, this indeed may be a direct effect of squelching. The observation (not shown) that a prolonged induction was able to recover growth capacity by further reducing the proportion of positive cells in those populations supported this assumption as the residual, transgene negative cells started to overgrow the transgene positive cells.
